# Supplementary material for: MicroRNA Profiling of PRELI-Modulated Exosomes and Effects on Hepatic Cancer Stem Cells
Source: Int J Mol Sci. 2024 Dec 11;25(24):13299. doi: 10.3390/ijms252413299 (PMC11678812; doi:10.3390/ijms252413299)

Concentration of induced Exosomes

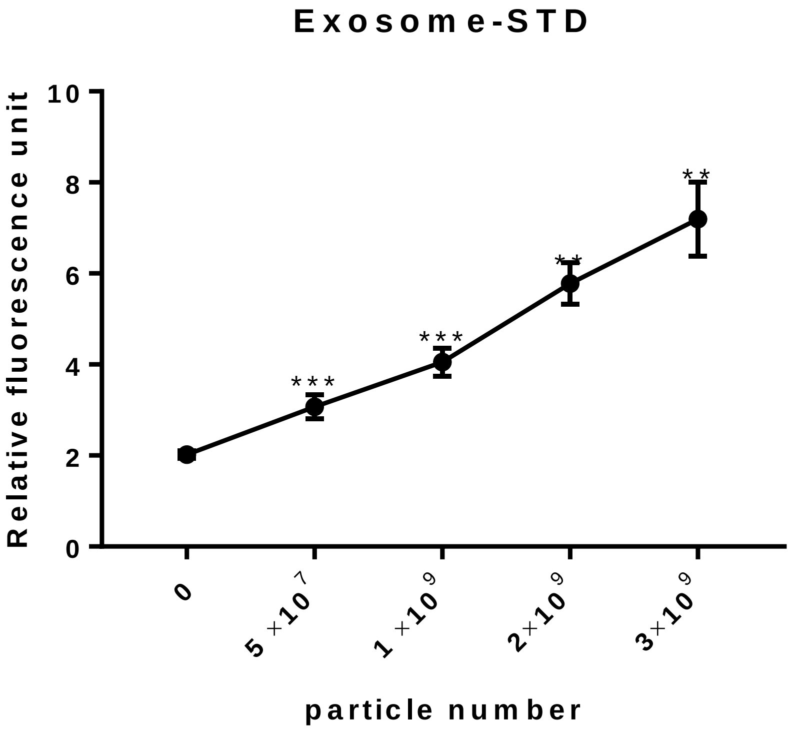

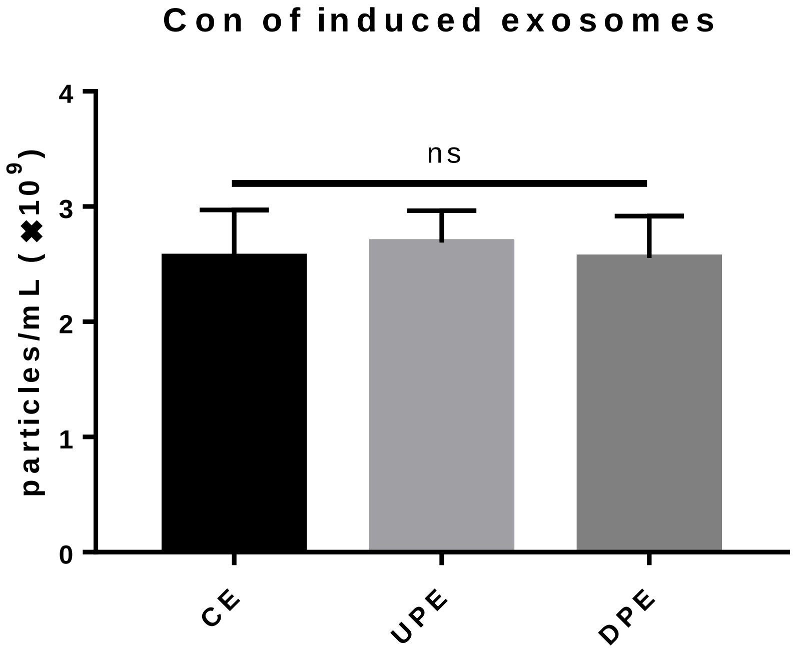

Cytotoxicity Concentration 50 (CC_{50}) of the induced exosomes

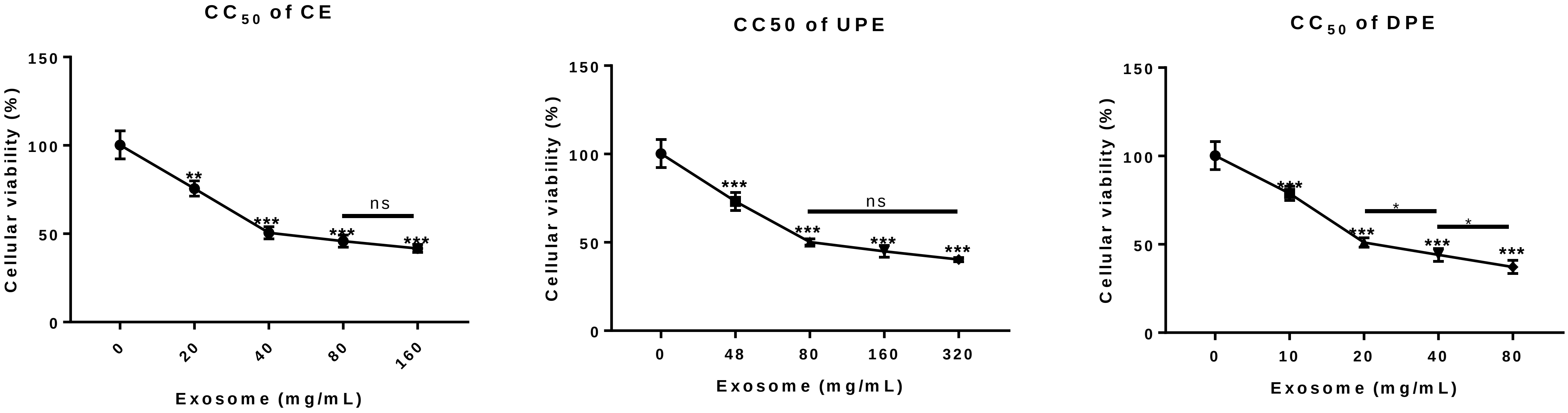

Western blotting membrane

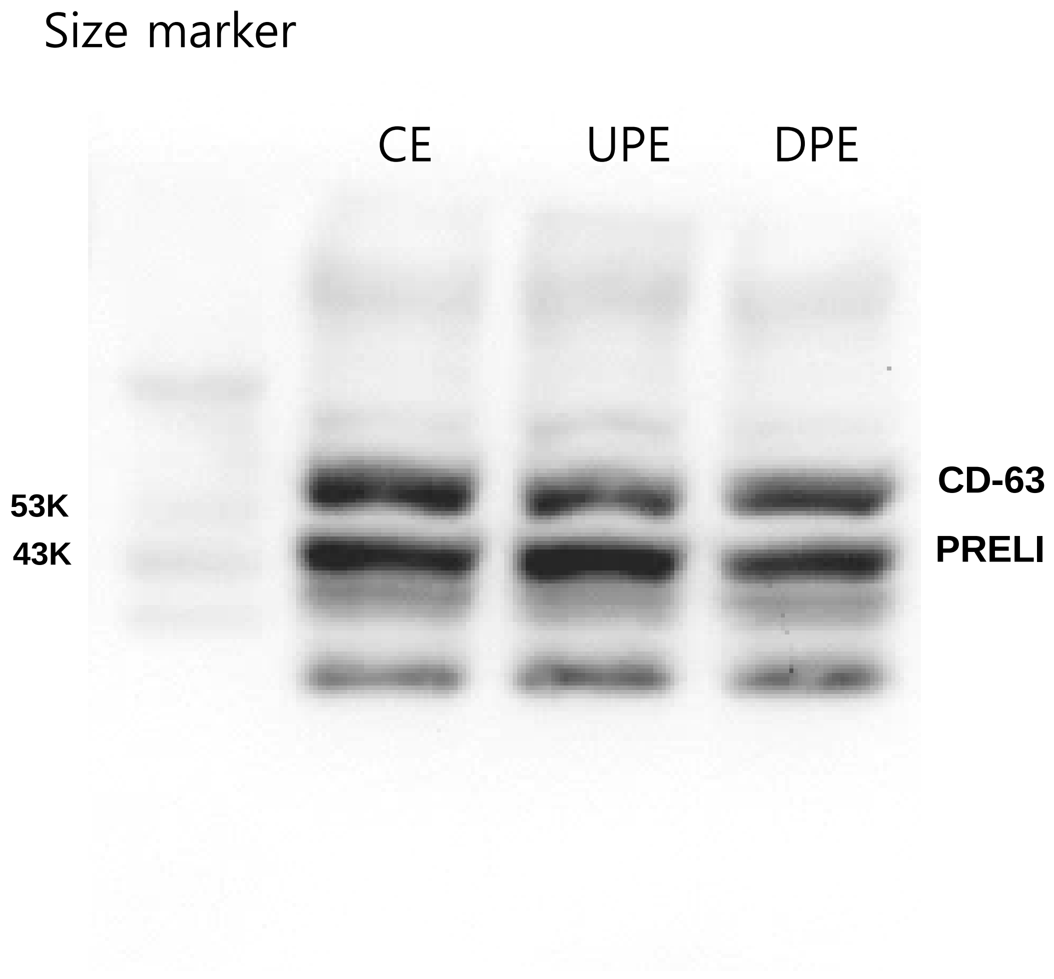

Supplement: Supplementary file 1 [file ijms-25-13299-s001.zip › ijms-3323759-supplementary.pdf]
